# A FRET-Based Assay for the Identification of PCNA Inhibitors

**DOI:** 10.3390/ijms241411858

**Published:** 2023-07-24

**Authors:** Sarah Hardebeck, Sebastian Schreiber, Annika Adick, Klaus Langer, Joachim Jose

**Affiliations:** 1 University of Münster, Institute of Pharmaceutical and Medicinal Chemistry, Pharmacampus, 48149 Münster, Germany; sarah.hardebeck@uni-muenster.de (S.H.);; 2 University of Münster, Institute for Pharmaceutical Technology and Biopharmacy, Pharmacampus, 48149 Münster, Germany; annika.adick@uni-muenster.de (A.A.); k.langer@uni-muenster.de (K.L.)

**Keywords:** PCNA, p15, FRET, inhibitor screening, ATLD2, disease, variant, stability, aggregation, free-energy calculations

## Abstract

Proliferating cell nuclear antigen (PCNA) is the key regulator of human DNA metabolism. One important interaction partner is p15, involved in DNA replication and repair. Targeting the PCNA–p15 interaction is a promising therapeutic strategy against cancer. Here, a Förster resonance energy transfer (FRET)-based assay for the analysis of the PCNA–p15 interaction was developed. Next to the application as screening tool for the identification and characterization of PCNA–p15 interaction inhibitors, the assay is also suitable for the investigation of mutation-induced changes in their affinity. This is particularly useful for analyzing disease associated PCNA or p15 variants at the molecular level. Recently, the PCNA variant C148S has been associated with Ataxia-telangiectasia-like disorder type 2 (ATLD2). ATLD2 is a neurodegenerative disease based on defects in DNA repair due to an impaired PCNA. Incubation time dependent FRET measurements indicated no effect on PCNA^C148S^–p15 affinity, but on PCNA stability. The impaired stability and increased aggregation behavior of PCNA^C148S^ was confirmed by intrinsic tryptophan fluorescence, differential scanning fluorimetry (DSF) and asymmetrical flow field-flow fractionation (AF4) measurements. The analysis of the disease associated PCNA variant demonstrated the versatility of the interaction assay as developed.

## 1. Introduction

Controlled DNA metabolism is crucial for cell survival. The key regulators in DNA metabolism are sliding clamps that encircle the DNA helix. Sliding clamps are ubiquitously present in all living organisms and in some viruses [1]. Despite their low sequence homology and varying oligomeric states, all sliding clamps show a high overall structure similarity with a pseudo six-fold symmetry [2,3,4,5]. They function as binding platform for proteins that are involved in DNA metabolism and tether them to the DNA at the replication fork. Additionally, sliding clamps increase the processivity of DNA polymerases [4,6].

Human DNA metabolism is controlled by the 86 kDa homotrimeric proliferating cell nuclear antigen (PCNA) [7,8]. Through the interaction with a variety of binding partners, PCNA is involved in DNA synthesis, replication, repair, chromatin assembly, chromatin maintenance and cell cycle control [8,9,10]. Many proteins bind PCNA via their PCNA-interacting protein (PIP)-box or PIP degron that targets PCNA for degradation. The binding occurs at the front face of PCNA between two domains of one subunit [11,12,13]. There are three binding sites for PIP-box interactions, one at each monomer. Protein binding is strongly regulated by post-translational modifications, binding affinities and local protein concentrations [10]. Other interaction motifs are the KA-box [14] or the AlkB homologue 2 PCNA-interacting motif (APIM), which overlap with the binding site of PIP-box proteins [15,16].

Due to the importance of PCNA for DNA metabolism, an altered function can cause drastic consequences for the cell. The expression of PCNA is elevated in many types of tumors and is associated with high proliferation rates [17,18,19,20,21]. Targeting PCNA by interfering with the PCNA interaction network, preventing trimerization or reduction in chromatin stability are promising therapeutic strategies. The clinical relevance of PCNA led to the identification of peptidic, small molecule, and aptamer inhibitors of PCNA interactions, which efficiently reduce cell growth and induce cell death [21,22]. A promising example of a PCNA interaction inhibitor is ATX-101, which is currently in phase II clinical studies. ATX-101 is a cell-penetrating peptide based on the APIM binding motif. It reduces primary metabolism, inhibits DNA repair, induces apoptosis and increases the effect of chemotherapeutic agents in many tumor types and cancer cell lines, while healthy cells were less affected [23,24,25]. Another example of a PCNA inhibitor targeting the PCNA binding groove is T2 amino alcohol (T2AA) [26]. T2AA disrupt the interaction of PCNA to PIP-box containing proteins and increases the effects of DNA damaging agents [26,27,28]. Molecules that bind to the hydrophobic binding pocket of PCNA affect a large network of protein–protein interactions in multiple signaling pathways. This can lead to undesired side effects. The inhibition of one specific therapeutically relevant interaction might circumvent this problem. The PCNA–p15 interaction is particularly well suited for this purpose [29]. p15 is an intrinsically disordered protein involved in DNA replication and repair by its interaction with PCNA [10]. Increased expression of p15 is associated with cancer and chemotherapeutic resistance [30,31,32,33]. p15 interacts with its canonical PIP-box sequence (^62^QKGIGEFF^69^) at the front site of PCNA. But what makes the p15 interaction special is that the p15 residues 59–62 form a β-turn, which directs the N-terminus of p15 towards the channel of the ring. This allows the interaction of the residues ^51^NPVCVRPT^58^ to the inner side of the PCNA ring [29]. The unique binding mode allows the specific targeting of the therapeutically relevant PCNA–p15 interaction.

Although an elevated PCNA expression correlates with highly proliferative cells and is associated with tumors, low PCNA numbers or an impaired functionality can also be fatal for the cell. PCNA knockout in mice is embryonically lethal [34], indicating the essential role of PCNA for the cell. It is hardly surprising that almost no disease associated PCNA mutations have been discovered so far. However, in 2014, the PCNA^S228I^ variant was linked to ataxia-telangiectasia-like disorder type 2 (ATLD2) [35]. ATLD2 is a neurodegenerative disorder with underlying defects in DNA repair based on an impaired PCNA. The main clinical features are telangiectasia, sensorineural hearing loss, premature aging, short stature, photosensitivity and neurodegeneration [35]. Further symptoms include developmental delay, foot deformity, speech and swallowing deficits, muscle weakness and mild oculomotor apraxia [35,36]. Previous studies showed that in patient-derived cells, an unchanged PCNA^S228I^ protein level and an unaffected DNA replication is observed. However, the cells show an enhanced sensitivity against UV radiation indicating defects in DNA repair [35]. Structural studies with PCNA^S228I^ observed a large conformational change at the interdomain connection loop (IDCL) and at the PIP-box binding groove [37], which likely impairs the interaction of several PIP-box containing proteins [35,37]. Recently, the PCNA variant NM_002592.2(PCNA): c.443G > C(p.C148S) was associated with ATDL2 [36]. The molecular consequences of this mutation were not understood during the experiments of this publication. In the meantime, however, a drastic decrease in the thermal stability of PCNA^C148S^ has been demonstrated [38].

In this study, the development of a FRET-based PCNA–p15 interaction assay is presented. The assay is capable of determining an accurate dissociation constant of the PCNA–p15 interaction and is suitable for the identification of inhibitors of the aforementioned interaction. We furthermore used the assay to characterize the PCNA^C148S^ mutation. No impairment of the binding affinity was evident. Free energy calculations, however, suggested a destabilizing effect of the mutation on PCNA. A reduced thermo- and chemical stability of the PCNA^C148S^ protein was shown by differential scanning fluorimetry (DSF) and intrinsic tryptophan fluorescence measurements. Asymmetrical flow field-flow fractionation (AF4) measurement also showed an increased aggregation of PCNA^C148S^. These results taken together suggest that PCNA^C14SS^ impairs the stability of the protein but has little to no influence on the direct interaction between PCNA and p15. These findings provide a first model on the molecular consequences that may underlie the ATDL2 phenotype associated with the PCNA^C148S^ mutation.

## 2. Results

### 2.1. Development of a FRET Assay to Analyze the PCNA–p15 Interaction

**Figure 1:**
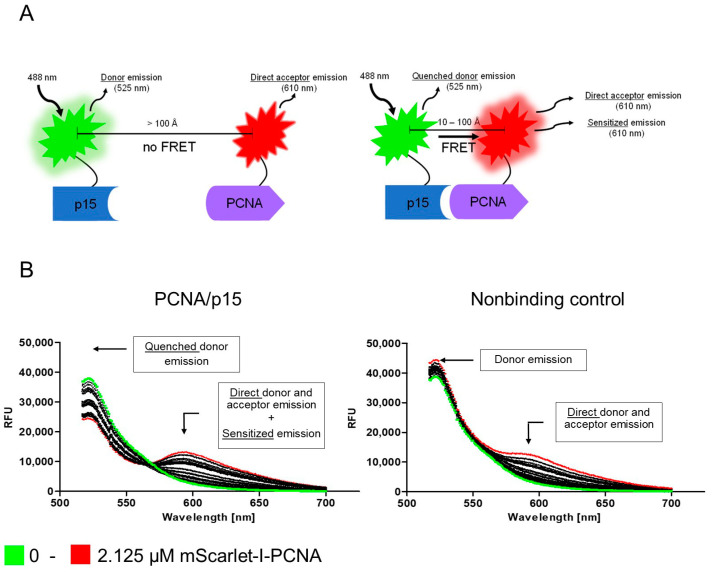
Analysis of PCNA–p15 interaction by FRET. (**A**) Schematic representation of the FRET-based assay for analysis of PCNA–p15 interaction. Full length PCNA was fused with mNeonGreen and p15 was fused with mScarlet-I. The PCNA–p15 interaction brings the fluorescent proteins mNeonGreen (donor) and mScarlet-I (acceptor) in close proximity. This allows energy transfer from the donor molecule to the acceptor, resulting in a reduced donor emission (donor quenching) and an increased acceptor emission (sensitized emission). Binding is evaluated by analyzing the sensitized emission using the three-channel method. (**B**) An amount of 1 µM p15-mNeonGreen (left) or mNeonGreen (right) was incubated with 0–2.125 µM mScarlet-I-PCNA for 1 h at 37 °C. The fluorescence was excited at 488 nm and the emission was detected at 517–700 nm. For p15-mNeonGreen/mScarlet-I, a quenched donor emission and an increased acceptor emission was observed. The nonbinding control showed a substantial amount of direct donor and acceptor emission at 595 nm. A three-channel method was used to compensate for this spectral crosstalk, as explained in the methods.

The PCNA–p15 interaction presents a promising target for cancer therapy. For screening PCNA–p15 inhibitors targeting the unique interaction site, a FRET-based binding assay was established. For this purpose, p15 and PCNA were genetically conjugated with a FRET donor-acceptor pair. The p15-mNeonGreen and mScarlet-I-PCNA fusion proteins were expressed in *E. coli* BL21(DE3) cells and purified by affinity chromatography afterwards. For analysis of PCNA–p15 interaction by FRET, 1 µM p15-mNeonGreen was mixed with varying concentrations of mScarlet-I-PCNA (0–2.125 µM) and incubated at 37 °C for 1 h. Fluorescence was then excited at 488 nm and the emission spectra were measured at 500–700 nm (Figure 1B). The detected emission maxima correspond to the emission of mNeonGreen at 517 nm and mScarlet-I at 594 nm [39,40]. Due to the wide excitation range of mScarlet-I, the acceptor is also directly excited by donor excitation. In addition, the emission spectrum of the donor overlaps with that of the acceptor. Accordingly, three emissions were detected for mScarlet-I-PCNA-p15-mNeonGreen when excited at 488 nm: direct donor molecule emission, direct acceptor molecule emission and sensitized emission. The emission at 594 nm is elevated for mScarlet-I-PCNA/mNeonGreen-p15 in comparison to the non-binding control (mNeonGreen/mScarlet-I-PCNA). Simultaneously, the donor emission is reduced with increasing acceptor concentrations (donor quenching). The occurrence of both observations proves the energy transfer from mNeonGreen to mScarlet-I upon PCNA–p15 interaction. Thus, the assay setup is suitable for quantitative analysis of PCNA–p15 interaction and inhibition.

**Figure 2:**
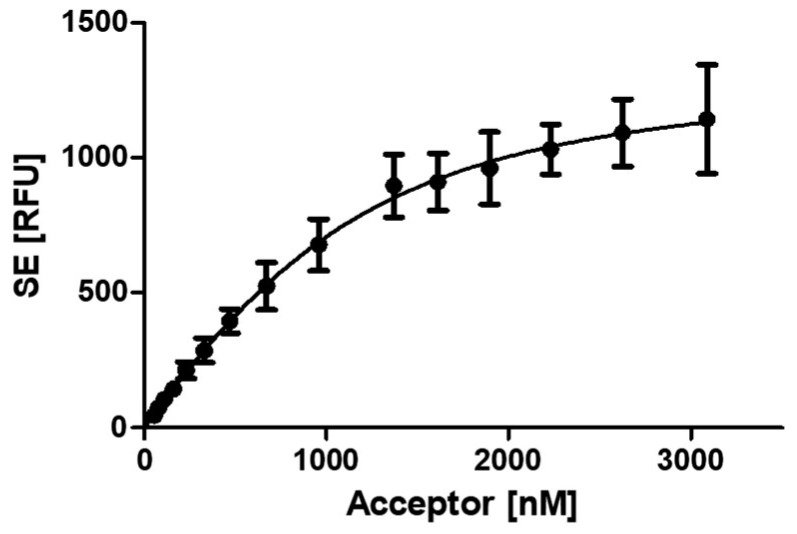
Affinity analysis of PCNA–p15 interaction by FRET. An amount of 1 µM p15-mNeonGreen was incubated with 0–3.09 µM mScarlet-I-PCNA for 1 h at 37 °C. Binding is evaluated by analyzing the sensitized emission using the three-channel method. The sensitized emission (SE) of three independent experiments in triplicates was plotted against the mScarlet-I-PCNA concentration, resulting in a K_D_ value of 430.8 ± 59.9 nM for the PCNA–p15 interaction. The given error is the standard error of the fit.

For the analysis of the PCNA–p15 affinity, the samples were prepared as in the experiment before. Binding was evaluated by analyzing the sensitized emission (SE) using the three-channel method, which considers the spectral crosstalk of the fluorophores [41,42]. By plotting the SE against the acceptor concentration, a dissociation constant of 430.8 ± 59.9 nM for the PCNA–p15 interaction was determined (Figure 2). This is in good agreement with the binding affinity already published for PCNA–p15 interaction (isothermal titration calorimetry, 1.1 µM) [29].

**Figure 3:**
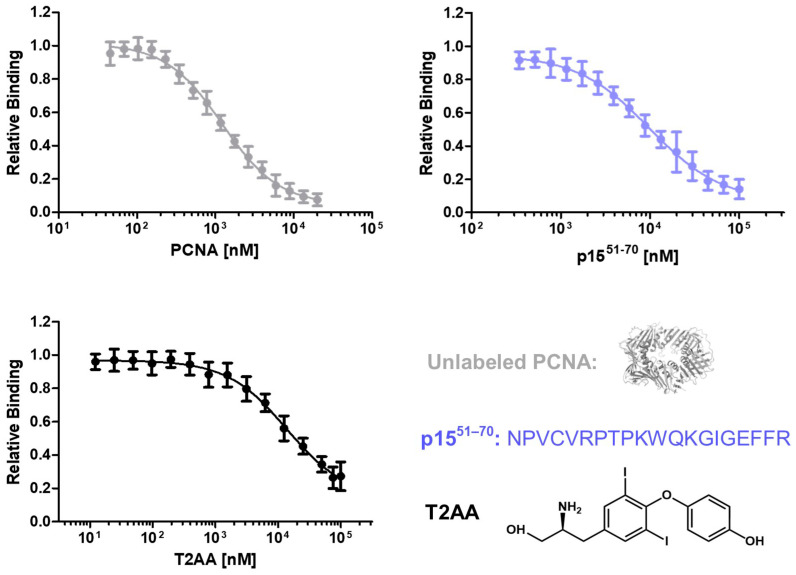
Analysis of the PCNA–p15 inhibition by unlabeled PCNA, peptide p15^51–70^ and small molecule inhibitor T2AA. An amount of 1 µM p15-mNeonGreen and 1 µM mScarlet-I-PCNA were incubated with varying inhibitor concentrations (0–100 µM for T2AA and p15^51–70^, 0–20 µM for unlabeled PCNA) for 1 h at 37 °C. Relative binding was plotted against PCNA, p15^51–70^ or T2AA concentrations, resulting in IC_50_ values of 1.24 ± 0.07, 9.81 ± 0.99 and 13.81 ± 2.0 µM, respectively. The given error is the standard error of the fit. K_i_ values were determined using Cheng–Prusoff equation [44] and turned out to be at 0.37 ± 0.03 (unlabeled PCNA), 2.94 ± 0.31 (p15^51–70^) and 4.12 ± 0.61 µM (T2AA), respectively.

To investigate the ability to detect inhibitory molecules in this assay, the effect of nonfluorescent PCNA, the peptide p15^51–70^ and the small molecule inhibitor T2AA were tested (Figure 3). Unlabeled PCNA competes with mScarlet-I-PCNA for the binding to p15-mNeonGreen. Therefore, a reduced binding signal of p15-mNeonGreen/mScarlet-I-PCNA in presence of unlabeled PCNA is expected. p15^51–70^ is a peptide based on the sequence of p15 which binds competitively to p15 at PCNA. T2AA is an already published PCNA inhibitor interacting with the PIP-box binding groove [26] and should inhibit the PCNA–p15 interaction. To test the inhibitory effect of the molecules, 1 µM mScarlet-I-PCNA and 1 µM p15-mNeonGreen were incubated with varying concentrations of the inhibitors. By plotting the inhibitor concentration against the sensitized emission, IC_50_ values of 1.23 ± 0.06 (unlabeled PCNA), 9.81 ± 0.99 (p15^51–70^) and 13.81 ± 2.0 µM (T2AA) were determined. K_i_ values were calculated using the Cheng Prusoff equation and turned out to be 0.38 ± 0.02 (unlabeled PCNA), 2.99 ± 0.26 (p15^51–70^) and 4.21 ± 0.55 µM (T2AA), respectively. Assuming that the conjugation of mScarlet-I does not affect the binding affinity of PCNA–p15, the expected K_i_ value is equal to the determined K_D_ value [43]. The observed K_D_ value of 0.43 µM is quite similar to the K_i_ value actually obtained in this assay, demonstrating the accuracy of the method. T2AA inhibits the interaction of the PIP-box containing PL-peptide with PCNA at an IC_50_ of 1–1.5 µM [27,28] and a K_i_ value of 0.91–1.36 µM was calculated. Thus, T2AA-induced inhibition of the PCNA–p15 interaction is in the same concentration range as the inhibition of the PCNA–PL peptide interaction. Accordingly, this assay is suitable for the identification and quantification of inhibitory peptides and small molecules targeting the PCNA–p15 interaction.

### 2.2. Screening for PCNA Inhibitors

To identify new classes of PCNA inhibitors, compounds of the in-house database were analyzed regarding their effect on the PCNA–p15 interaction. Due to the absence of a clear structural idea, they were randomly selected from a series of pyranobezochinones, aminomethylfuropyranones, totarols and benzofuran-4,7-diones to identify a hit. However, no promising results were obtained. Due to the limited size and chemical diversity of the in-house substance library, only a few different scaffolds were tested. It is, therefore, unfortunate but not unlikely that a small-scale screen, as performed here, does not yield a molecule with inhibitory activity. Taking into account that typical hit rates in classical high-throughput screenings are below 1%, this appears reasonable [45]. A larger screening of a more diverse set of compounds could lead to the identification of an active compound, with inhibitory activity on the PCNA–p15 interaction.

### 2.3. Analyzing the Relevance of the Mutation C148S for PCNA Function

**Figure 4:**
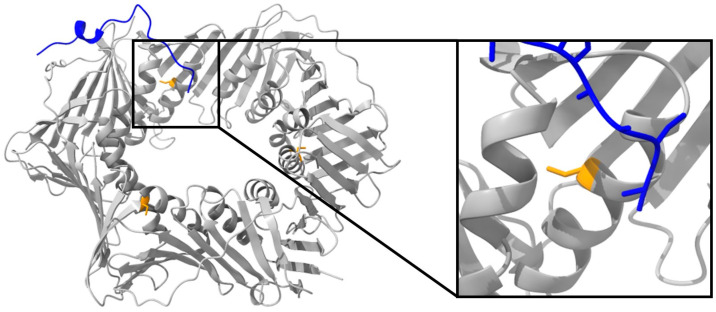
Structure of human PCNA in complex with p15^52–71^ (PDB code 6EHT). Human PCNA is shown in gray with the position of the mutation at C148 highlighted in orange. p15^52–71^ is presented in blue and interacts with its C-terminal site with the hydrophobic pocket of PCNA. The N-terminal site binds to the inner site of the PCNA ring near the position of the mutation C148S. The position of the C148S mutation is emphasized in the window.

Next to the application as inhibitor screening tool, the FRET assay was also accurate in determining the binding affinity of PCNA–p15. Thus, mutational effects on this interaction can be investigated. Recently, the mutation C148S was associated with the disease ATLD2 [36,38]. However, nothing was known about the consequences of this mutation for PCNA function. C148 is highly conserved in different eukaryotic species (Table S1), indicating the importance of the cysteine at position 148 for PCNA. C148S is located at the inner site of the PCNA ring, far away from the typical binding site of most PCNA interaction partners (Figure 4). Its neighboring amino acid R149 is involved in sliding of PCNA along the DNA helix as well as for p15 binding [29,46]. We employed the established FRET assay to determine the influence of the C148S mutation on the interaction between PCNA and p15.

#### 2.3.1. C148S Reduces the Apparent Binding of PCNA to p15

**Figure 5:**
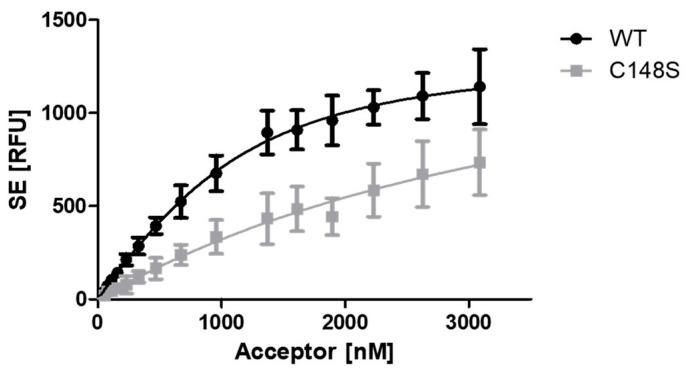
Analysis of the effect of C148S on the PCNA–p15 binding by FRET. An amount of 1 µM p15-mNeonGreen was incubated with 0–3.09 µM mScarlet-I-PCNA (black circles) or mScarlet-I-PCNA^C148S^ (grey squares) for 1 h at 37 °C. The sensitized emission (SE) of three independent experiments was plotted against the acceptor concentrations, resulting in K_D_ values of 0.43 ± 0.06 µM for PCNA^WT^ and 3.09 ± 1.11 µM for PCNA^C148S^. The given error is the standard error of the fit. RFU = relative fluorescence units.

The influence of C148S on PCNA–p15 binding was analyzed by the FRET assay (Figure 5). p15-mNeonGreen was used as donor, and varying concentrations of mScarlet-I-PCNA^WT^ or mScarlet-I-PCNA^C148S^ were used as acceptor molecule. The sensitized emission was plotted against the acceptor concentration and K_D_ values of 0.43 ± 0.06 µM for p15/PCNA^WT^ and 3.09 ± 1.11 µM for p15/PCNA^C148S^ were ascertained. Thus, an approximately 7-times lower apparent binding of PCNA^C148S^ in comparison to the wild-type protein was observed. However, saturation must be approached to accurately determine K_D_ values from saturation binding experiments [47]. This was not the case for the p15/PCNA^C148S^ interaction, resulting in an uncertainty of the obtained K_D_ value. Nevertheless, it can be concluded that the incorporation of C148S strongly impairs the apparent binding of PCNA with p15.

#### 2.3.2. Alchemical Free-Energy Calculations Predict a Destabilizing Effect of the C148S Mutation on PCNA

We next asked if the reduced apparent binding signal was caused by a direct impairment of the p15/PCNA binding site or by an affected structure or stability of PCNA. Therefore, we conducted alchemical free energy calculations. Alchemical free-energy calculations are a useful in silico method for studying the effect of mutations, as they can provide remarkable prediction accuracy, if properly set up [48,49]. It is possible to estimate the differences in the free energy (ΔG) between two defined sets of states, i.e., the unfolded and folded state of a protein or the bound and free state of a complex. The effect of mutations on this free energy difference can also be given as the relative change in ΔG, which is commonly referred to as the relative folding free energy (ΔΔG_folding_) or the relative binding free energy (ΔΔG_affinity_). In the case of PCNA, this allows to study the effect of the C148S mutation on protein stability (ΔΔG_folding_) and PCNA–p15 affinity (ΔΔG_affinity_).

Alchemical free-energy methods work by gradually transforming one chemical state into another following an unphysical pathway. The alchemical pathway does not need to follow the physical folding or binding pathway and is, therefore, more accessible by molecular dynamics (MD) simulations. In our case, we made use of a non-equilibrium approach based on the Jarzynski equality and the Crooks fluctuation theorem [50,51,52]. In this approach, two standard MD simulations are run, one for each state (WT and C148S) of the system. Snapshots from these equilibrium simulations are extracted and used to perform alchemical transition by driving the Hamiltonian *H* of the system from one state to another using a parameter called λ. This parameter is gradually increased from 0 to 1 during the simulation.

**Figure 6:**
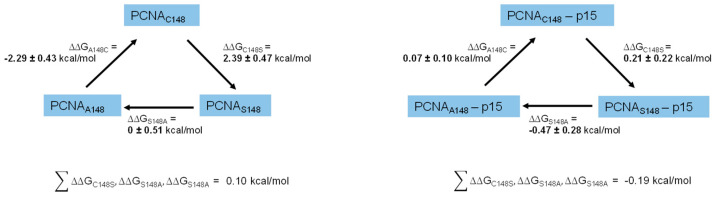
Closed thermodynamic cycles of the ΔΔG_folding_ of PCNA (**A**) and ΔΔG_affinity_ of PCNA–p15 (**B**) upon amino acid mutation. ΔΔG values were obtained using the thermodynamic cycle shown in the methods section. The sum of all ΔΔG values within one cycle was calculated and resulted in values close to zero for both experiments, implicating good convergence of the simulations. The calculated ΔΔG for the mutation PCNA^C148S^ suggest a destabilizing effect of the mutation on PCNA (ΔΔG_C148S_ = 2.39 kcal/mol). The same mutation is predicted to have no effects on the binding free energy between PCNA and p15 (ΔΔG_C148S_ = 0.21 kcal/mol). The uncertainty is the standard deviation of three independent replicas.

To estimate both the ΔΔG_folding_ of PCNA and the ΔΔG_affinity_ between PCNA and p15 upon amino acid mutation, the wild-type amino acid was alchemically morphed from a cysteine at position 148, to a serine. To keep the systems to a reasonable size, we included only one PCNA monomer, instead of the homotrimer. This drastically reduced computational cost. The stability of monomeric PCNA during 100 ns of MD simulation was tested. No great conformational change, and a root mean square deviation (RMSD) below 3 Å for the protein was observed. We therefore concluded that it was viable to only use the monomer going onward. For the calculation of ΔΔG_folding_ the transition for the unbound state, in this case approximated by a Gly-Cys-Gly tripeptide, and the folded state were performed [53]. For the calculation of ΔΔG_affinity_, the transitions were performed for free PCNA and the PCNA–p15 complex. The resulting ΔG values for each transition were used to calculate the ΔΔG values according to the thermodynamic cycle shown in Figure S4. We also performed calculations for two further mutations to construct a closed thermodynamic cycle with the respective ΔΔG values at its edges to confirm convergence of the simulations (Figure 6). This cycle includes mutations for serine to alanine and alanine to cysteine, forming a cycle with three edges. Furthermore, the simulation length for the transitions were optimized by running these simulations with different transition times. This optimization was performed for the mutation involving the biggest perturbation, in this case, A148C. ΔΔG_folding_ was monitored as a function of transition time and no significant change in ΔΔG_folding_ was observed when increasing the transition time beyond 100 ps (Figure S5). We therefore concluded that a transition time of 100 ps was sufficient for the mutations and the system under investigation. Following this optimized setup, cycle closure for the calculation of ΔΔG_folding_ and ΔΔG_affinity_ was achieved, with all the single legs of each cycle summing up to 0.1 kcal/mol and −0.19 kcal/mol. These values are within the expected range of error for alchemical free energy calculations [48,49], and within the uncertainty of our approach as seen by the standard deviation over three independent replicas. The estimated ΔΔG_folding_ value of 2.39 ± 0.47 kcal/mol for the mutation C148S suggests a destabilizing effect of the mutation. An increase in ΔΔG_folding_ is equivalent to a more positive folding free energy of PCNA^C148S^. This leads to a decreased thermostability and/or chemical stability [54]. It furthermore increases the probability of the protein to adapt intermediate folding states, which are normally poorly populated. These intermediate states often display large hydrophobic patches on their surface which can initiate aggregation [55]. The calculated ΔΔG_affinity_ of 0.21 ± 0.22 kcal/mol for the C148S mutation suggests no direct change in binding affinity of PCNA^C148S^ to p15 compared to PCNA^WT^. One likely conclusion is that the mutation leads to a less stable variant of PCNA, which might be more prone to aggregation or denaturation. When this protein is used as the titrator in a binding assay, less functional protein than expected might be present in solution. The measured affinity would, therefore, be apparently lower than it actually is. This might explain the apparently reduced binding of p15 to PCNA measured with the FRET assay, even if the binding free energy is not changed by the mutation. We therefore decided to experimentally test the thermal and chemical stability of PCNA^C148S^, as well as its aggregation behavior.

#### 2.3.3. The Amino Acid Substitution C148S Reduces the Chemical Stability of PCNA

**Figure 7:**
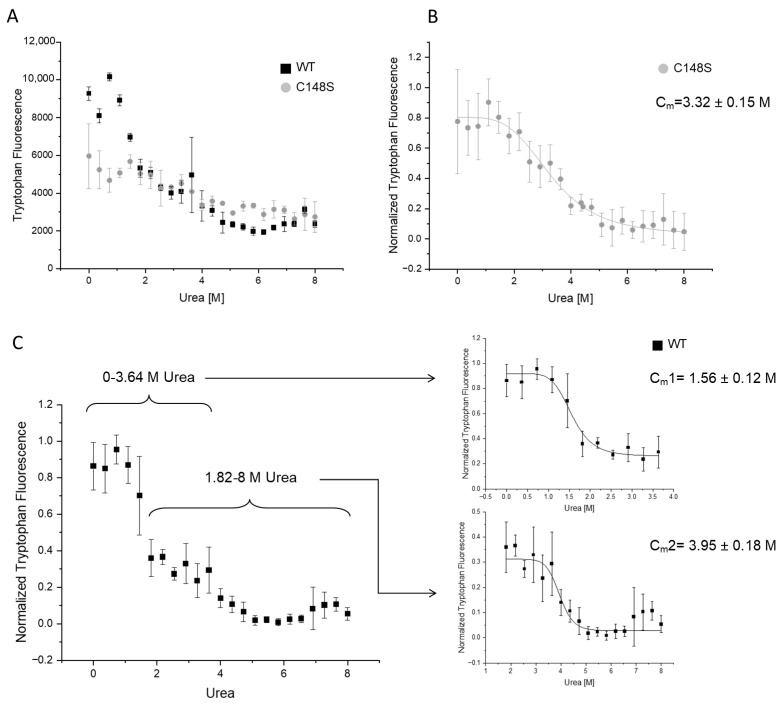
Analyzing the influence of C148S on the chemical stability of PCNA by intrinsic tryptophan fluorescence measurements. The urea-induced denaturations of PCNA^WT^ (black) and PCNA^C148S^ (grey) were measured by analyzing the intrinsic tryptophan fluorescence at 320 nm. (**A**) The fluorescence intensity of PCNA was plotted against varying urea concentrations (0–8 M) in an overlay of one representative experiment. (**B**) The normalized tryptophan fluorescence of PCNA^C148S^ of three experiments was plotted against the urea concentration. PCNA^C148S^ denatures at a C_m_ of 3.32 ± 0.15 M urea. (**C**) The normalized tryptophan fluorescence of PCNA^WT^ of three experiments was plotted against the urea concentration. Due to the three-state denaturation of PCNA^WT^, the graph was split into two parts for the determination of the C_m_ values. A C_m_1 at 1.56 ± 0.12 M and a C_m_2 at 3.95 ± 0.18 M urea were ascertained. The standard deviation of C_m_ is the standard error of the fit.

For analyzing the influence of C148S on the chemical stability of PCNA, we measured the intrinsic tryptophan fluorescence at 320 nm (Figure 7). The PCNA trimer contains three tryptophans (W28), one per monomer, allowing the analysis of the chemical stability by the intrinsic tryptophan fluorescence. The tryptophans of PCNA are buried in a hydrophobic environment, and become more exposed to the aqueous solvent during unfolding. The aqueous environment results in a blue-shifted tryptophan emission, reducing the emission at 320 nm [56].

Our results show a three-state behavior for the urea-induced denaturation of the wild-type protein. Such a three-state curve indicates the presence of an intermediate state where the tryptophans are more exposed to the solvent. In comparison to the wild-type PCNA, the denaturation of PCNA^C148S^ arises in only two states. The overlay of both curves reveals a high similarity of the tryptophan fluorescence of PCNA^WT^ and PCNA^C148S^ between 2 and 8 M urea (Figure 7A). Since the intrinsic fluorescence of tryptophans is strongly dependent on their environment, the tryptophans (W28) probably undergo similar conformational states between 2 and 8 M urea in both proteins.

Without the addition of urea, the tryptophan fluorescence is higher for PCNA^WT^ than for PCNA^C148S^. Accordingly, the tryptophans are probably more buried in the wild-type protein under this condition. The tryptophan fluorescence intensity of PCNA^C148S^ is constant between 0 and 2 M urea and similar to the fluorescence intensity of the wild-type’s intermediate state. One possible explanation might be that PCNA^C148S^ predominantly populates this intermediate conformational state between 0 and 2 M urea, even in the absence of urea. Based on these results, a decrease in chemical stability due to the incorporation of C148S was concluded.

Furthermore, the midpoint denaturations (C_m_) for the complete denaturation were determined, which were at 3.95 ± 0.18 M for PCNA^WT^ and at 3.32 ± 0.15 M for PCNA^C148S^. Accordingly, the higher C_m_ value of PCNA^C148S^ and the different denaturation states indicate a decrease in PCNA stability due to the incorporation of C148S.

#### 2.3.4. C148S Affects the Thermal Stability of PCNA

The intrinsic tryptophan fluorescence measurements revealed a negative effect of C148S on the stability of PCNA. To confirm this result, the thermal stability of PCNA was analyzed by differential scanning fluorimetry (DSF). In the assay, an extrinsic fluorescent dye is used whose fluorescence is quenched in an aqueous environment. Upon protein unfolding, more hydrophobic sites are accessible for the interaction with the fluorescent dye, resulting in an elevated fluorescence intensity. This allows the analysis of thermal protein denaturation based on the fluorescence intensity of the dye.

**Figure 8:**
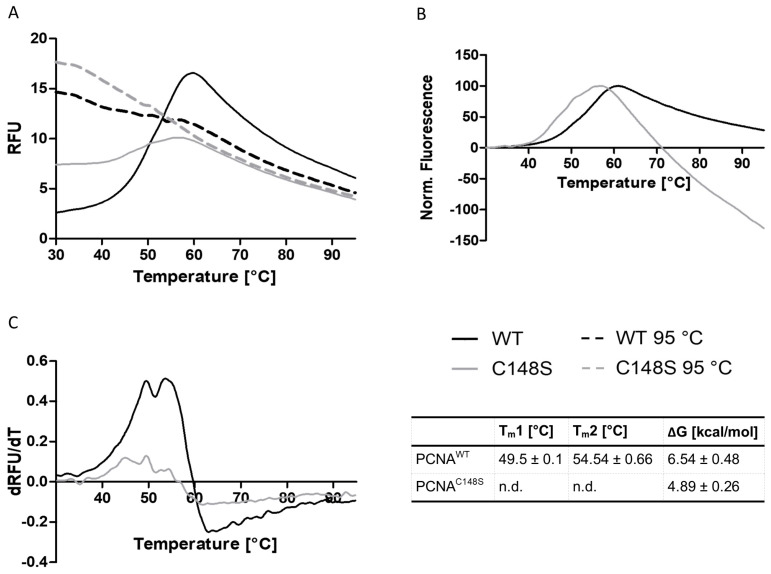
Analyzing the influence of C148S on the thermal stability of PCNA by DSF. (**A**) Representative melting curves of PCNA^WT^ (black line), PCNA^C148S^ (grey line), denatured PCNA^WT^ (black dashed line) and denatured PCNA^C148S^ (grey dashed line). (**B**) Normalized melting curves of PCNA^WT^ and PCNA^C148S^ indicate a reduced thermal stability of PCNA^C148S^. (**C**) First derivative of the melting curve of PCNA^WT^ and PCNA^C148S^. For PCNA^WT^ two melting points were determined, one at 49.5 ± 0.1 °C and one at 54.54 ± 0.66 °C. The calculated ΔG values are 5.65 ± 0.48 kcal/mol for PCNA^WT^ and 4.89 ± 0.26 kcal/mol for PCNA^C148S^.

The DSF experiment revealed aberrations in the denaturation profile of PCNA^C148S^ and PCNA^WT^ (Figure 8). PCNA^C148S^ shows a higher initial fluorescence signal in comparison to PCNA^WT^, which implies more hydrophobic patches on the exposed surface of PCNA^C148S^ prior to heating. Such exposed hydrophobic patches might be explained by denatured or unproperly folded protein samples [57]. This observation is consistent with the interpretation of the previous experiment, where we assumed a stronger exposure of W28 to the aqueous solvent for PCNA^C148S^ than for PCNA^WT^.

The fluorescence intensity of PCNA^C148S^ peaks at lower temperatures in the normalized plot, indicating the denaturation of PCNA^C148S^ at lower temperatures (Figure 8B). By plotting the first derivative of the melting curves against the temperature, two melting points for PCNA^WT^ (T_m1_ = 49.1 ± 0.1 and T_m2_ = 54.54 ± 0.66 °C) were determined. Accordingly, similar to the previously performed chemical denaturation, a three-state denaturation profile for PCNA^WT^ was detected. Due to the poor resolution of the PCNA^C148S^’s derivative, the determination of T_m_-values was not possible here.

ΔG-values were estimated from the DSF melting curves, as explained in detail in the methods section. A ΔG-value for protein unfolding of 6.54 ± 0.48 kcal/mol for PCNA^WT^ and 4.89 ± 0.26 kcal/mol for PCNA^C148S^ was ascertained. Thus, the experimentally determined ΔΔ***G****_unfolding_* (1.65 ± 0.74 kcal/mol) is similar to the ΔΔ***G****_unfolding_* values predicted by the alchemical free energy calculations (2.39 ± 0.47 kcal/mol). The measured positive ΔΔ***G****_unfolding_* value as well as the normalized melting curves strongly supports our hypothesis that C148S impairs the thermal stability of PCNA.

#### 2.3.5. PCNA^C148S^ Exhibits an Enhanced Aggregation Behavior

**Figure 9:**
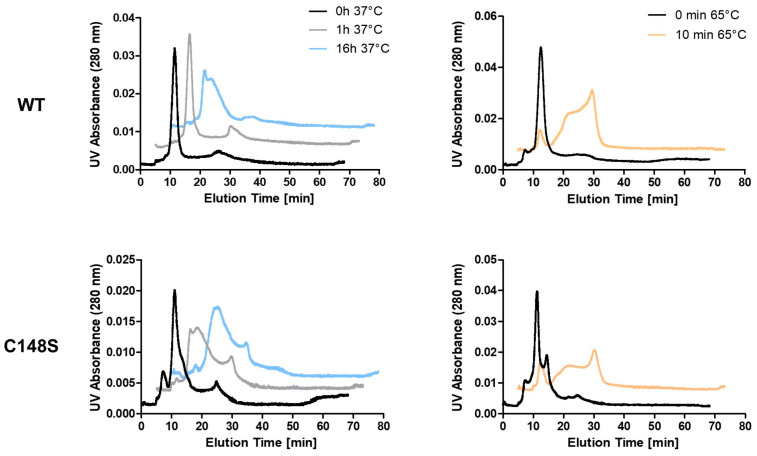
Analysis of the aggregation behavior of PCNA by AF4. Proteins were incubated at 37 °C for 0 h (black), 1 h (grey) or 16 h (blue) or at 65 °C for 10 min (orange), and were injected to AF4 afterwards. The induction of aggregates was evaluated by plotting the UV absorbance at 280 nm against the elution time. The increase in thermal stress correlates with elevated elution times, which can be explained by the formation of higher molecular weight complexes.

Reduced protein stability leads to more frequent folding/unfolding intermediates with exposed hydrophobic sites on the protein surface. This can initiate the formation of protein aggregates. Reduced protein stability is, therefore, often also associated with an increased aggregation behavior [55]. Thus, we asked whether the introduction of the mutation C148S also has an influence on the aggregation behavior of PCNA. The use of asymmetrical flow field-flow fractionation (AF4) enables the separation of proteins based on their hydrodynamic diameter and is suitable to detect protein aggregates [58]. Aggregation was induced by incubating PCNA^WT^ or PCNA^C148S^ at 37 °C for 0, 1, or 16 h or at 65 °C for 10 min (Figure 9). The molecular weight of the samples was estimated using the co-running size standard.

Based on the analysis of the molecular weight standard, both PCNA^WT^ and PCNA^C148S^ were mainly present in their trimeric forms after 0 h at 37 °C (elution time around 10–15 min). These data are consistent with crosslinking experiments, where trimers for both PCNA^WT^ and PCNA^C148S^ were detected (Figure S3). When comparing PCNA^WT^ and PCNA^C148S^ after 0 h at 37 °C, an additional early elution peak (elution time around 5–10 min) is more pronounced for PCNA^C148S^, which might correlate to monomeric PCNA.

High temperatures induce protein unfolding and often promotes aggregation [59]. Since a T_m_-value of 54.54 °C was determined for PCNA^WT^ unfolding by DSF, we induced the aggregation of PCNA at 65 °C for 10 min. This condition resulted in later molecule elution times (above 15 min) and broader signal peaks and was used as positive control for protein aggregation (Figure 9). In AF4, later elution times correlate with higher hydrodynamic diameters of the molecules. Increasing the thermal stress by elongation of the incubation time at 37 °C elevated the protein elution time (above 15 min) for both, PCNA^WT^ and PCNA^C148S^. This increase in the elution time occurred more rapidly for PCNA^C148S^ than for the PCNA^WT^, which is particularly evident after protein incubation at 37 °C for 1 h (Figure 9). These high hydrodynamic diameter molecules can be explained by the formation of protein aggregates.

Accordingly, PCNA^C148S^ exhibits a lower thermal stability and an enhanced aggregation behavior. Since both PCNA^WT^ and PCNA^C148S^ were mainly present in their trimeric form after 0 h at 37 °C, we suggest that the mutation does not affect the trimerization of the protein.

#### 2.3.6. The Impaired Thermal Stability of PCNA^C148S^ Is Responsible for Its Reduced Binding Signal to p15 Measured by FRET

The prediction of ΔΔG_affinity_ suggests no impairment of PCNA^C148S^ affinity towards p15. The method proved to be quite accurate in predicting the ΔΔ***G****_unfolding_*. However, a higher K_D_-value for p15 binding to PCNA^C148S^ in comparison to PCNA^WT^ was determined. We therefore decided to test whether this discrepancy might be a result of reduced protein stability. The PCNA binding assays include an incubation step at 37 °C for 1 h. AF4 measurements revealed that such protein incubation elevated the hydrodynamic diameter of PCNA^C148S^. In contrast, the hydrodynamic diameter of PCNA^WT^ remained almost unchanged. This is also true for mScarlet-I-PCNA^WT^ and mScarlet-I-PCNA^C148S^ (Figure S2), which were used for the FRET bindings assay. Accordingly, a lower amount of mScarlet-I-PCNA^C148S^ might be available for the binding assays, which might affect the apparent binding.

**Figure 10:**
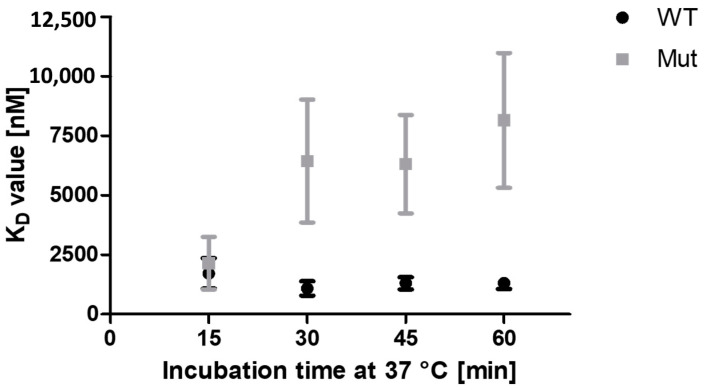
Incubation time dependent analysis of PCNA–p15 affinity. The affinities of 1 µM p15 to PCNA^WT^ or PCNA^C148S^ (0–3.09 µM) were determined by FRET assay after 15, 30, 45 and 60 min incubation at 37 °C. The K_D_-values of PCNA^WT^ are constant at different incubation times. The K_D_-values of the PCNA^C148S^/p15 interaction increases during the incubation at 37 °C, indicating the stability caused decrease in the apparent binding.

To check this assumption, FRET measurements were performed, in which the affinities were determined after varying incubation times at 37 °C (Figure 10). The K_D_-values of mScarlet-I-PCNA^WT^ and mScarlet-I-PCNA^C148S^ are similar after 15 min at 37 °C. However, after 30 min incubation, the K_D_-values of mScarlet-I-PCNA^C148S^ to p15-mNeonGreen are increased, while the affinity of mScarlet-I-PCNA^WT^ to p15-mNeonGreen remains constant over 60 min. The consistent K_D_-values of mScarlet-I-PCNA^WT^ indicate that the binding of p15 and PCNA is already in equilibrium after 15 min incubation. The dramatic increase in the K_D_-values of mScarlet-I-PCNA^C148S^ from 30 min at 37 °C suggests an effect of the incubation on the calculated binding affinities. Based on the lower stability of PCNA^C148S^, we conclude that the continued incubation at 37 °C results in less functional mScarlet-I-PCNA^C148S^ that is available for p15-mNeonGreen binding. These results strengthen our confidence that the measured decreased binding affinity of PCNA^C148S^ after 60 min at 37 °C is caused by the impaired thermal stability of PCNA^C148S^.

## 3. Discussion

### 3.1. Development of a FRET-Based Assay to Analyze the PCNA–p15 Interaction

In this study, a FRET-based assay for the identification of specific PCNA–p15 inhibitors was developed. This assay is a convenient method for analyzing the effect of proteins, peptides and small molecules on the PCNA–p15 interaction. The determined binding affinity is in the same range as the already published K_D_ value of PCNA and p15 measured by isothermal titration calorimetry (1.1 µM) [33], indicating the accuracy of the method. However, as the p15-mNeonGreen concentration is similar to the dissociation constant, a titration effect on the determined K_D_ value cannot be excluded [60].

The assay was performed in 384 well plates and can easily be scaled up to a 1536 format [61]. The conjugation with fluorescent proteins facilitates protein purification, obviates the need for additional labeling steps, and can increases protein stability and, thus, the robustness of the assay. In addition, fluorescent fusion proteins are more environmentally friendly in comparison to other hazards such as radioisotopes. The PCNA–p15 FRET assay is similar to fluorescence polarization methods, that are already reported for screening of PCNA-PIP-box inhibitors [27,28,62,63]. However, the FRET assay can be applied using plate readers with simpler optics that are widely distributed in many laboratories. The assay focusses on the in vitro characterization of PCNA–p15 interaction inhibitors. A FRET-based in vivo assay between the PCNA–APIM interaction is already known for HeLa cells [24]. An analogous PCNA–p15 interaction assay might provide further information about the effect of potential inhibitors inside the cell. In addition to the identification of PIP-box binders, this also allows the identification of specific inhibitors of the PCNA–p15 interaction. These specific inhibitors may circumvent undesired side effects due to interference with various DNA metabolism processes. However, the FRET assay does not allow the direct differentiation between PCNA-PIP box inhibitors and specific PCNA–p15. This question can be investigated by testing the inhibitory activity of a promising compound for further PIP-box binding partners.

### 3.2. The Amino Acid Substitution C148S Reduces the Amount of Functional PCNA

In addition to the application as screening tool for PCNA inhibitors, we also successfully used the FRET assay for analysis of mutational effects on PCNA–p15 binding. In this study, we investigated the functional consequences of the newly-described PCNA mutation C148S, which is related to ATLD2. Although we initially assumed an effect of the C148S incorporation on PCNA–p15 binding, we now suggest that the mutation does not affect PCNA–p15 affinity. This conclusion is consistent with the recently published data of Magrino et al., in which no impairment of the binding affinity of PCNA^C148S^ to PIP-box interaction partners was detected [38]. The incubation time dependent analysis of the K_D_-values and the free energy calculation for PCNA–p15 affinity indicate that the previously measured decreased binding of PCNA^C148S^ is rather related to a reduced amount of natively folded PCNA^C148S^ than to its lower affinity. When a protein with a reduced stability is used as the titrator in a binding assay and incubated for 1 h at 37 °C, less functional protein is present in solution. The measured affinity would, therefore, be apparently lower than it actually is. The incubation time dependent FRET measurements support this hypothesis as the K_D_ value of PCNA^WT^ was constant over 1 h, while the K_D_ value of PCNA^C148S^ increased after continuous incubation at 37 °C. The decreased amount of functional PCNA can be explained by the reduced chemical and thermal stability of PCNA^C148S^, which was also confirmed by Magrino et al. [38]. Destabilizing mutations are more likely to have intermediate folding states with external hydrophobic regions, which are normally marginally populated. An increased frequency of an intermediate state of PCNA^C148S^ was indicated by the chemical denaturation experiment. There, W28 of PCNA^C148S^ was predominantly present in the intermediate conformation even without the addition of urea. Further, the high initial fluorescence values of PCNA^C148S^ in the DSF measurement also indicated an increased exposure of hydrophobic amino acids on the protein surface. Such external hydrophobic sites often initiate protein aggregation [55,64], explaining the dramatically increased aggregation behavior of PCNA^C148S^ measured by AF4. However, the affinity measurements, the detection of PCNA^C148S^-trimers by AF4 and the unchanged crystal structure of PCNA^C148S^ prove that the intermediate PCNA state is at least partially functional.

Now, the question arises why a substitution of cysteine to serine impairs the stability of PCNA. PCNA does not exert any stabilizing disulfide bonds [65] that could be prevented by the amino acid exchange. However, it has been shown that a cysteine to serine substitution reduces the stability and increases the aggregation behavior of proteins even if they are not involved in disulfide bonds [66,67]. C148 is buried in a hydrophobic environment and its sulfur atom forms a Π-bond with the backbone of F144 [38,65]. Although there is a high structure similarity between serine and cysteine, buried cysteines are more hydrophobic, which might explain the destabilizing effect of serine at this position. The free energy calculations indicate that the exchange to both serine and alanine destabilizes PCNA. Accordingly, the highly conserved cysteine at position 148 is important for PCNA stability.

### 3.3. Consequences of the Reduced PCNA^C148S^ Stability for the Cell

The functional consequences of PCNA^C148S^ for the cell are not fully understood yet. Based on our data and the reduced amount of chromatin bound PCNA^C148S^ in patient-derived cells [38], we are suggesting that the mutation is hypomorphic in nature. PCNA downregulation causes drastic consequences for the cell. It abolishes DNA synthesis and cell proliferation and impairs the genomic integrity and DNA repair [68,69,70]. However, it is unlikely that PCNA^C148S^ causes such strong effects, because it would probably not be compatible with life. To date, only one other human disease-related PCNA variant has been described, which also underlies ATLD2 [35]. The common component that unites both ATLD2 associated PCNA variants is the dramatic loss of the stability [38]. The two ATLD2 associated PCNA variants PCNA^S228I^ and PCNA^C148S^ differ with respect to their binding affinities to PIP-box binding partner. The impairment of the binding affinity of PCNA^S228I^ is probably caused by the altered structure of PCNA^S228I^, while the structure of PCNA is not affected by C148S incorporation [37,38]. PCNA^S228I^ has no effect on DNA replication, but on DNA repair [35]. PCNA and its binding partners are present at high levels during DNA replication [71,72]. Green et al. suggested that the high level of PCNA at the replication fork might compensate the reduced binding affinity of PCNA^S228I^ during DNA replication [73]. It could be similar for PCNA^C148S^. The lower stability of PCNA^C148S^ might be less important during DNA replication due to its high expression level. The effect of the C148S amino acid substitution might also be fatal for DNA repair. Considering that symptoms of ATLD2 patients are already linked with defects in DNA repair [35], it might be reasonable that the C148S also affects DNA repair. The impact of C148S on DNA repair was already suggested by the higher sensitivity of patient cells to IR- or zeocine-induced DNA damage [38].

Beyond that, the increased aggregation behavior of PCNA^C148S^ might contribute to the disease. In addition to the loss of protein function, aggregation can affect cellular protein homeostasis [74]. Many disease-associated protein variants show an increased aggregation, indicating the relevance of aggregation on various human diseases [75]. Future work on analyzing the effects of PCNA aggregation on the cells might give further insights for understanding of the disease.

### 3.4. PCNA Stabilizing Molecules as Therapeutic Option

In accordance with Magrino et al. [38], we suspect that the disease is essentially driven by the lower stability of PCNA^C148S^. A possible therapeutic approach might be the identification of a PCNA stabilizing molecule. Such pharmaceutical chaperones continue gaining importance for the treatment of destabilizing missense mutations [76,77]. Due to the quantities of PCNA interaction partners [8,9], a stabilizer might interfere with the interaction of other binding partners. However, most PCNA interaction partners bind at similar sites at the front site of PCNA [13,14,15]. Accordingly, the identification of an allosteric stabilizer might be a promising approach.

## 4. Materials and Methods

### 4.1. Plasmid Construction and Mutagenesis

A list of all used oligonucleotides is given in Table S2. The construct PET28b-PCNA, pET11d-p15 has been kindly provided by Alfredo de Biasio (King Abdulla University of Science and Technology, Thuwal, Saudi Arabia). For the generation of the p15-mNeonGreen plasmid, the sequence of mNeonGreen from *Branchiostoma lanceolatum* [40] was integrated C-terminal of the p15 sequence, connected by a flexible linker (GGGGS). The incorporation of the mNeonGreen sequence was performed by In-Fusion Cloning (Clontech Laboratories, Takara, Saint- Germain-en-Laye, France). Analogously, the sequence of mScarlet-I [39] was fused to the N-terminus of PCNA for the construction of the mScarlet-I-PCNA expression plasmid. mScarlet-I and PCNA were connected by the hydrophilic flexible linker (GEGQGQGPGRGYAYRS) [78]. The C148S substitution was introduced in the PCNA sequence by site directed mutagenesis.

### 4.2. Protein Purification

*E. coli* BL21(DE3) cells containing the corresponding plasmid were cultivated at 37 °C to an optical density (OD_578nm_) of 0.6. For induction of PCNA or mScarlet-I-PCNA expression, 1 mM IPTG was added and the cells were incubated for 16 h at 23 °C. After cultivation, the cell pellet was resuspended in buffer A (50 mM Tris-HCl, pH 7.6, 150 mM NaCl, 10% glycerol, 1 mM benzamidine, 1 mM PMSF). Cells were disrupted by sonification and the solution was centrifuged at 50,000× *g* for 1 h at 4 °C afterwards. The lysate was added to the nickel NTA column, previously equilibrated with buffer B (50 mM Tris-HCl, pH 7.6, 150 mM NaCl, 10% glycerol). The protein elution was induced by increasing concentrations of imidazole in buffer B (50–500 mM). The quality of the purification was analyzed by SDS-PAGE and Coomassie brilliant-blue staining. The PCNA containing fractions of interest were dialyzed against phosphate-buffered saline (PBS), aliquoted, frozen with liquid nitrogen and stored at −80 °C.

The expression of p15-mNeonGreen was induced by the addition of 1 mM IPTG and the cells were incubated at 30 °C for additional 4 h. The cell pellet was resuspended in buffer C (20 mM Tris-HCl, pH 8, 150 mM NaCl, 10% glycerol, 1 mM benzamidine, 1 mM PMSF) and the cells were disrupted by sonification on ice. The solution was centrifuged at 50,000× *g* for 1 h at 4 °C and the supernatant was transferred to a column equilibrated with buffer D (20 mM Tris-HCl, pH 8, 150 mM NaCl, 10% glycerol). p15-mNeonGreen was eluted with increasing concentrations of imidazole (50–500 mM) in buffer D. The quality of the purification was analyzed by SDS-PAGE and Coomassie brilliant-blue staining. The protein fractions of interest were dialyzed against PBS containing 1 mM DTT, aliquoted, frozen with liquid nitrogen and stored at −80 °C.

The protein concentration of p15-mNeonGreen was determined using a nanophotometer (Pearl, Implen GmbH, Munich, Germany) and the extinction coefficient 52,830 M^−1^cm^−1^ at 280 nm. The concentration of PCNA was determined as promoter concentration using the extinction coefficient at 280 nm (15,930 M^−1^cm^−1^ for His-PCNA and 53,290 M^−1^cm^−1^ for His-mScarlet-I-PCNA).

### 4.3. FRET Binding Assay

For the FRET-K_D_-assay, 1 µM p15-mNeonGreen and variable concentrations of mScarlet-I-PCNA (0–3.1 µM) were mixed in PBS containing 1 mM DTT. For initial testing of inhibitors, 1 µM p15-mNeonGreen, 1 µM mScarlet-I-PCNA and 50 µM of the inhibitors were used. Sensitized emission and donor quenching were used to evaluate the inhibitory activity of the compounds. Only substances that reduced the binding signal in both analytical methods were defined as inhibitors of the PCNA–p15 interaction. IC_50_ values were determined using 1 µM p15-mNeonGreen, 1 µM mScarlet-I-PCNA and varying inhibitor concentrations. The assay was performed in a final volume of 20 µL per well in a 384 well microtiter plate (3766, Corning, NY, USA). After incubation at 37 °C for 60 min at 300 rpm, the fluorescence of the samples was detected using the microplate reader infinite 200Pro (Tecan, Männedorf, Switzerland). The fluorescence was measured in three channels: 488/525 nm, 561/610 nm and 488/610 nm and the FRET effect was determined by evaluating the sensitized emission [41]. mNeonGreen as well as mScarlet-I were used as non-binding controls, respectively (Figure S1). The sensitized emission of mNeonGreen/mScarlet-I-PCNA was subtracted from the sensitive emission of the p15-mNeonGreen/mScarlet-I-PCNA interaction. For the determination of the binding affinities, the sensitized emission of three experiments was measured in technical triplicates and plotted against the acceptor concentration. The K_D_-values were calculated using following K_D_-Fit [41] (Equation (1)) and GraphPad Prism 5 (GraphPad Software, La Jolla, CA, USA):

(1) $$Em_{FRET}=Em_{FRETmax}\left(1-\frac{2K_{D}}{X-a+K_{D}+\sqrt{{\left(X-a-K_{D}\right)}^{2}+4K_{D}X}}\right)$$

where ***Em****_FRET_* is the *FRET* signal intensity, ***Em****_FRETmax_* is the maximum of ***Em****_FRET_* and *a* is the constant donor concentration.

For the determination of the inhibitory activities, the sensitized emission of three experiments was measured in technical triplicates, normalized and plotted against the inhibitor concentration. The IC_50_-values were calculated using the log (inhibitor) vs. response- variable slope (four parameters) of GraphPad Prism 5.0.2 for Windows (GraphPad Software, Boston, Massachusetts USA):

The small molecule positive control T2AA was purchased from Sigma Aldrich (St. Louis, MO, USA). The peptide p15^51–70^ containing the amino acids NPVCVRPTPKWQKGIGEFFR was commercially acquired from GenicBio Limited (Shanghai, China).

### 4.4. Differential Scanning Fluorimetry (DSF)

The thermal stability of PCNA^WT^ and PCNA^C148S^ was determined by DSF [79]. An amount of 4 µM PCNA was mixed with 20 × SYPRO Orange Protein Gel Stain (Sigma Aldrich, St. Louis, USA) in PBS. The samples were heated from 30 to 95 °C by 0.5 °C/5 s in the Rotor-Gene Q 2 Plex HRM (Qiagen, Hilden, Germany). SYPRO orange was excited at 470 ± 10 nm and the emission was detected at 610 ± 5 nm. The measurement was conducted three times in quadruplicates. The melting points were obtained using the Rotor-Gene Q software by determining the extreme points of the first derivative of the fluorescence dF/dT(T) formed from the melting curves as function F(T). Δ***G****_unfolding_* was estimated from the melting curves with the method described by Wright et al. [80]. In short, the fraction of folded protein, *P_f_*, at any given temperature is calculated with Equation (2), where ***F_min_*** is the minimal measured fluorescence, ***F_max_*** is the maximal fluorescence and ***F*** is the measured fluorescence at the given temperature.

(2) $$P_{f}=1-\frac{F-F_{min}}{F_{max}-F_{min}}$$

Next, the fraction of unfolded protein, ***P_u_***, is calculated with Equation (3) at each temperature.

(3) $$P_{u}=1-P_{f}$$

***K_u_***, the equlibrium constant of unfolding, is calculated with Equation (4).

(4) $$K_{u}=\frac{P_{u}}{P_{f}}$$

The natural logarithm of ***K_u_*** was then plotted against 1/***T***, for all values corresponding to the protein folding between 10 and 50% with ***T***, being the absolute temperature. From the linear fit, Δ***H_unfolding_*** and Δ***S_unfolding_*** can be derived, with Δ***H_unfolding_*** being the slope, and Δ***S_unfolding_*** being the y intercept. Δ***G_unfolding_*** is then calculated using Equation (5).

(5) $$\Delta G_{unfolding}=\Delta H_{unfolding}-T\Delta S_{unfolding}$$

### 4.5. Asymmetrical Flow Field-Flow Fractionation (AF4)

AF4 was used for analysis of the aggregation behaviour of PCNA^WT^ and PCNA^C148S^. Thermal stress was induced by incubating the proteins at 37 °C for 1 or 16 h or at 65 °C for 15 min. Afterwards, the samples were cooled to 4 °C until they were injected into AF4. The AF2000 Flow FFF system (Postnova Analytics, Landsberg, Germany) coupled with an UV/Vis detector as well as a fluorescence detector (Shimadzu, Kyōto, Japan) was used. The UV/Vis detector was set to a wavelength of 280 nm. The fluorescence detector was set to an excitation wavelength of 569 nm and emission wavelength of 610 nm with a gain of 128. The Bio-Rad size standard (MW 1.35–670 kDa, Bio-Rad Laboratories, Hercules, CA, USA) served for the determination of the molecular weight of the analyzed proteins. The separation channel was equipped with a regenerated cellulose membrane (MWCO 10 kDa) and a trapezoidal spacer with tapered ends (height: 350 µm). All analyses were performed at 25 °C in phosphate buffer pH 6.6 (1.675 g/L NaH_2_PO_4_ × 1 H_2_O, 0.875 g/L Na_2_HPO_4_ × 2 H_2_O, 8.765 g/L NaCl) as mobile phase and samples were eluted at a detector flow rate of 0.5 mL/min. The samples (injection volume of 20 µL) were injected into the separation channel during focusing step (4 min) at an injection flow of 0.2 mL/min, a cross-flow of 2.5 mL/min and a focus-flow of 2.8 mL/min. After a transition time of 0.2 min, the elution step was started: the cross-flow was kept constant (2.5 mL/min) for 10 min and then decreased linearly over 10 min to 0 mL/min. To ensure complete elution of the samples, the elution step was continued for 40 min with a detector flow of 0.5 mL/min (cross flow 0 mL/min). This was followed by a rinsing step with a detector flow of 0.2 mL/min (4 min). The data were evaluated using GraphPad Prism 5 (GraphPad Software, La Jolla, CA, USA) and a baseline correction was performed for a better data visualization.

### 4.6. Intrinsic Tryptophan Fluorescence

The intrinsic tryptophan fluorescence measurements were carried out in PBS containing 20 µL of 0.2 mg/mL PCNA^WT^ or PCNA^C148S^ solution in a 384 well microplate (788876, Greiner Bio-One, Kremsmünster, Austria). Chemical denaturation was induced by increasing urea concentrations (0–8 M) and a subsequent incubation at RT for 5 h. The intrinsic tryptophan fluorescence was excited at 284 nm and the emission were detected at 320 nm with a bandwidth of 9 nm at RT by using the plate reader Synergy MX (BioTek Instruments GmbH, Bad Friedrichshall, Germany). The normalized fluorescence intensities of three measurements in duplicates were plotted against the urea concentration. The C_m_-values were determined by using Equation (6) and OriginPro 2023 (OriginLab Corporation, Northampton, MA, USA), where ***A***2 is the final value, ***A***1 is the initial value, ***x***0 is the center and ***p*** is the power.

(6) y=A2+(A1−A2)/(1+(x/x0)^p)

Due to the biphasic denaturation profile of PCNA^WT^, the graph was split into two graphs for the determination of the C_m_-values. The given deviation of the C_m_ values is the standard error of the fit.

### 4.7. Alchemical Free Energy Predictions

#### 4.7.1. System Preparation

The crystal structure of PCNA in complex with p15 (PDB ID: 6GWS) was retrieved from the PDB server. Only one PCNA monomer in complex with p15 (chain A and chain D, respectively) was kept for all further calculations. The remaining structure was checked for missing atoms using the Structure Preparation panel from MOE (Molecular Operating Environment, Chemical Computing Group, Canada version: 2022.02). Protonation states were assigned with the Protonate 3D function using default settings at pH = 7.0. Mutations were generated using the Protein Builder from MOE. Sidechains were repacked and minimized using the Minimize tool, while tethering the backbone. The structures were exported from MOE in PDB format and used for MD system preparation within GROMACS 2019.3 [81].

#### 4.7.2. Free Energy Calculations and Hybrid Topologies

To estimate the relative free energy difference for the folding of PCNA or the binding of p15 to PCNA, we used a well-established thermodynamic cycle shown in Figure S4 [82]. The amino acids were morphed along the ΔG_1_ and ΔG_2_ arrows for the unfolded and folded, or the free PCNA and PCNA–p15 complex. The unfolded state for the estimation of the free folding energy difference, ΔΔG_folding_, was modelled by a glycine-x-glycine tripeptide, as described earlier [48,83]. All simulations were conducted separately. Hybrid topologies were generated using the pmx package after running the equilibrium simulations [83]. This way, the equilibrium trajectories can be used to extract coordinates for different transitions later on.

#### 4.7.3. MD Simulations

All MD simulations were carried out using GROMACS 2019.3. Proteins were modelled with the Amber99SB*ILDN forcefield [84], while water molecules were represented by the TIP3P water model [84]. Proteins, or tripeptides were solvated in a cubic box with 150 mM of Na^+^ and Cl^−^ ions. The total charge of the system was neutralized with either Na^+^ or Cl^−^. The system was energy minimized using the steepest decent algorithm, then equilibrated for 500 ps in the NVT ensemble and then for 500 ps in the NPT ensemble. Production simulations were run for 100 ns in the NPT ensemble. During simulation, H-Bonds were constrained using LINCS algorithm [85]. Particle Mesh Ewald was used to treat Electrostatic interactions in the simulation [86]. The cutoff for electrostatic interactions was set to 1.2 nm. Simulation temperature was controlled by the velocity rescaling thermostat at 300 K with a time constant of 0.1 ps [87]. Pressure coupling was performed using the Parrinello–Rahman barostat at 1 atm [88].

From these equilibrium trajectories, snapshots were extracted to construct hybrid topologies and perform the fast-alchemical switching reactions. The first 5 ns of every trajectory were discarded, and the rest was used to generate 95 snapshots (one every 1000 ps). Hybrid structures and topologies were than generated using the pmx package for each frame [83]. After energy minimization and 50 ps of equilibration, alchemical transitions were performed in 100 ps. This resulted in a change in λ value of 5 × 10^−5^/step. A softcore potential was used for the electrostatic interaction and the Lennard–Jones interactions during the transition, with default parameters. The work values for each transition were extracted and used to estimate the free energy at 300 K using the scripts provided with the pmx package. The free energy estimation was based on the Crooks Fluctuation Theorem [50]. Bennett acceptance ratio was used as a maximum likelihood estimator [89]. The uncertainty given is the standard deviation of the free energy estimation over three independent replicas.

### 4.8. Glutaraldehyde Crosslinking of PCNA

An amount of 46 µM PCNA^WT^ or PCNA^C148S^ was incubated with 0.004% glutaraldehyde in PBS for 10 min at RT. The reaction was stopped by the addition of 0.1 M glycine. For analysis of the crosslinking, 5 µg protein were loaded onto the SDS-PAGE gel (10%), followed by a Coomassie brilliant- blue staining or a western blot analysis.

### 4.9. Western Blot Analysis

The same volume of SDS sample buffer (100 mM Tris-HCl, 200 mM dithiothreitol, 4 % (*w*/*v*) SDS, 0.2 % (*w*/*v*) bromophenol blue, 20 % (*v*/*v*) glycerol, pH 6.8) was added to the protein samples and heated for 10 min at 95 °C. The proteins were separated by SDS PAGE and transferred to a polyvinylidene fluoride membrane using semidry electroblotting (Trans-Blot SD, Bio-Rad, Hercules, CA, USA). Afterwards, the membrane was blocked with 5% BSA in PBS over night at 4 °C under shaking. The immunodetection was conducted using first a His_6_ antibody (1:1000 in PBS with 0.01% NaN3, 1 h at RT, mouse anti-6x-His, Thermo Fisher Scientific, Waltham, MA, USA), followed by a horseradish peroxidase (HRP) coupled antibody (1:6000 in PBS with 0.1% Tween20, 1.5 h at RT, rabbit anti-mouse, Thermo Fisher Scientific, Waltham, USA). The washed membrane was treated with Immuno Cruz Western Blot Luminol Reagent (Santa Cruz Biotechnology, Dallas, TX, USA) and the luminicence was detected by chemiluminescence reader (ChemoCam ECL imager, Intas, Göttingen, Germany).

## Data Availability

Data are available in the manuscript and in the supplementary material as provided.

## Supplementary Materials

The following are available online at https://www.mdpi.com/article/10.3390/ijms241411858/s1.
