# Supplementary material for: A FRET-Based Assay for the Identification of PCNA Inhibitors
Source: Int J Mol Sci. 2023 Jul 24;24(14):11858. doi: 10.3390/ijms241411858 (PMC10380293; doi:10.3390/ijms241411858)
Supplement: Supplementary file 1 [file ijms-24-11858-s001.zip › ijms-2517157-supplementary.pdf]

Supplementary Materials:

Table S1. List of PCNA$^{141-155}$ amino acid sequences from different eukaryotic species using HomoloGene database [87]. Cysteine at position 148 was marked in red.

Organism	Sequence
Homo sapiens	SGEFARICRDLSHIG
Pan troglodytes	SGEFARICRDLSHIG
Macaca mulatta	SGEFARICRDLSHIG
Canis lupus familiaris	SGEFARICRDLSHIG
Bos taurus	SGEFARICRDLSHIG
Mus musculus	SGEFARICRDLSHIG
Rattus norvegicus	SGEFARICRDLSHIG
Gallus gallus	SAEFARICRDLSHIG
Xenopus tropicalis	SGEFARICRDLSHIG
Danio rerio	AMEFARICRDLAQFS
Drosophila melanogaster	AMEFARICRDLSQFG
Anopheles gambiae str. PEST	AGEFQKTCKDLSTFS
Caenorhabditis elegans	SSEFSKIVRDLSQLS
Saccharomyces cerevisiae S288C	SADFAKTVRDLSQLS
Kluyveromyces lactis	SAEFAKIIRDLNQLS
Eremothecium gossypii ATCC 10895	AAEFQRITRDLLTLS
Schizosaccharomyces pombe	SAEFRRICTDLLAMS
Pyricularia oryzae 70-15	SSEFKRITTDLMAMS
Neurospora crassa OR74A	SGEFSRICKDLSSIG
Arabidopsis thaliana	SNEFSRICKDLSSIG
Oryza sativa Japonica Group	SGEFARICRDLSQIG

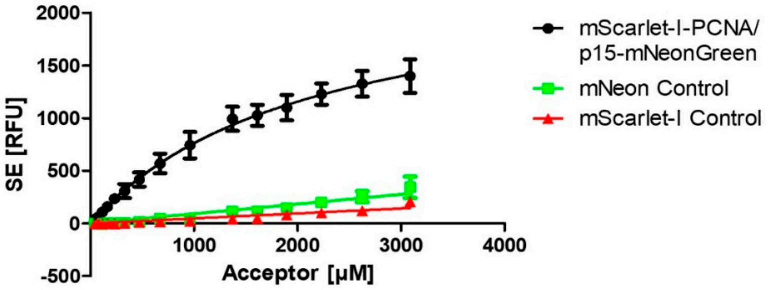

Figure S1. Proof of specific binding in the FRET assay. An amount of 1 µM of p15-mNeonGreen was incubated with increasing mScarlet-I-PCNA^{WT} concentrations (0-2.1 µM) for 1 h at 37 °C at 300 rpm. As nonbinding controls, the interaction of mNeonGreen/mScarlet-I-PCNA or mNeonGreen-p15/mScarlet-I was ~~analysed~~analyzed. The sensitized emission (SE) of three experiments in triplicates was shown. For further analysis of binding, the sensitized emission of the mNeonGreen control was subtracted from the sensitized emission of the samples. RFU= Relative Fluorescence Units.

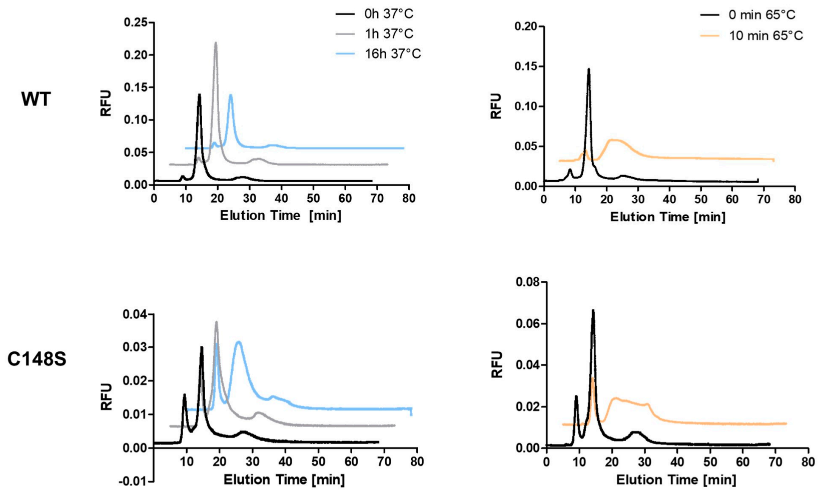

Figure S2. The C148S mutation decreases the thermal stability of mScarlet-I-PCNA. Proteins were incubated at 37 °C for 0 h (black), 1 h (grey) or 16 h (blue) or at 65 °C for 10 min (orange) and were afterwards injected to AF4. The induction of aggregates was evaluated by plotting the fluorescence emission at 610 nm against the elution time. The increase of thermal stress correlates with higher elution times, which can be explained by the formation of higher molecular weight complexes.

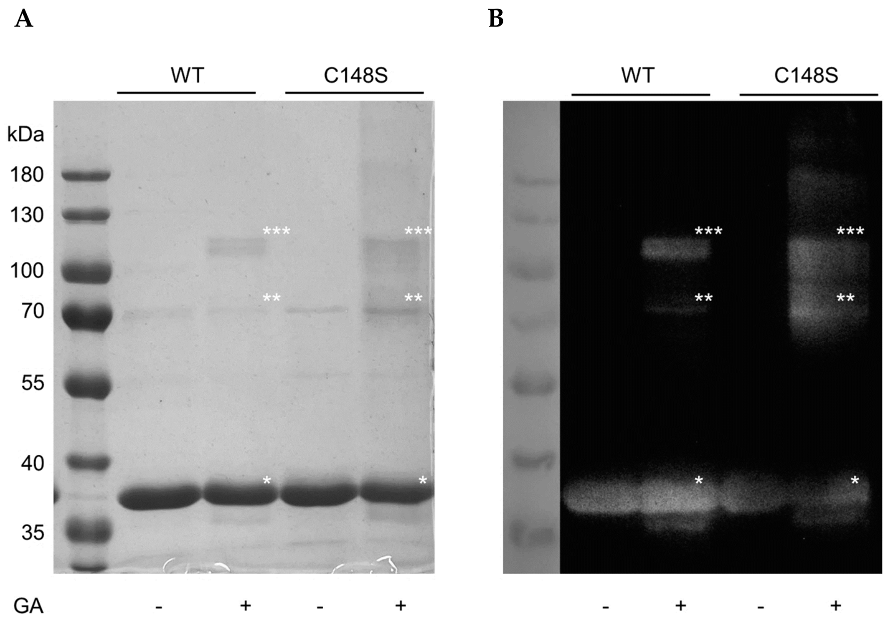

Figure S3. C148S does not prevent trimerization of PCNA. $PCNA^{WT}$ and $PCNA^{C148S}$ were crosslinked using 0.004% glutaraldehyde for 10 min at RT. 5 µg protein were loaded on a 10% SDS-PAGE gel and stained by Coomassie brilliant-blue **(A)** or transferred to an PVDF membrane and immunoblotted with an antibody against the His_6-epitope **(B)**. The asterisks mark the bands of the monomeric (*, 31.2 kDa) dimeric (**, 62.4 kDa) or trimeric (***, 93.6 kDa) PCNA.

Table S2. List of oligonucleotides used in this work.

**Number**	**Oligonucleotide sequence**	**Plasmid**	**Usage**
Pr2844 Pr2845	AGATCCACTCCACCCTCTT-TCTCATCGTTGGTG; GAGC-TTACAAGTAAGGATCCGG-CTGCTAAC	pSR052–vector	In-Fusion: generation of p15-mNeon intracellular expression plasmid
Pr1878 PR1879	GGTGAGGTGATCTATGGT-GACAAGGGCGAG; TTACT-TACAGCTCGTCCAT-GC	pSR052–insert	In-Fusion: generation of p15-mNeon intracellular expression plasmid
Pr427 Pr2837	CATATGATGGTGATGGTG-GTCATGGTATATCTCCTTC; GGTGGAGGTGGATCTATG-TTCGAGGCG	PSR046–vector	In-Fusion: generation of mScarlet-I-PCNA connected by the flexible linker (GGGGS).

Number	Oligonucleotide sequence	Plasmid	Usage
Pr1963<br>Pr2119	CACCATCACCATCAATGG-TGACAAGGGCGAGGC; AG-ATCCATCCACCCTGTACA-GCTCGTCCATGCC	pSR046-insert	In-Fusion: generation of mScarlet-I-PCNA connected by the flexible linker (GGGGS).
Pr1518<br>Pr2840	ATGTTCGAGGCGCGCCTGG; CTTACAGCTCGTCCATGC-CGC	pSR051–vector	In-Fusion: Incorporation of the hydrophilic flexible linker in mScarlet-I-PCNA sequence (GEGQGQGPGR GYAYRS)
Pr2838<br>Pr2839	GACGAGCTGTACAAGGGC-GAAGGCCAGGGCCAGGG-CCAGGGCCCGGGCCGCGG-CTATGCGTATCGCAGCAT-GTTCGAGGCGCGC; GCGC-GCCTCGAACATG	pSR051-Insert	In-Fusion: Incorporation of the hydrophilic flexible linker in mScarlet-I-PCNA sequence (GEGQGQGPGR GYAYRS)
Pr3199<br>Pr3200	TGAGATCTCGGGATATACGT GCAAATTCACC; TTTGC-ACGTATATCCCGAGATCT-CAGCC	pSR080; pSR081	Mutagenesis: Incorporation of C148S in PCNA or mScarlet-I-PCNA sequence

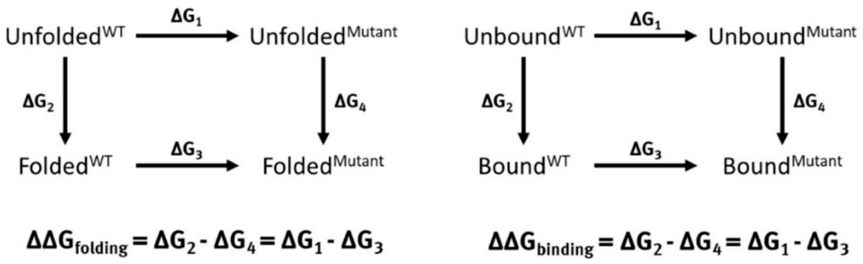

Figure S24. Thermodynamic cycle used for the calculation of $\Delta\Delta G_{folding}$ (left) and $\Delta\Delta G_{binding}$ (right). Amino acids were morphed along the non-physical path indicated by horizontal arrows to estimate the corresponding ΔG values. The ΔΔG value for a given mutation can then be obtained by the formula shown under the cycle. Vertical arrows indicate the physical folding or binding pathway. Obtaining the ΔG for these paths is considerably more computationally expensive.

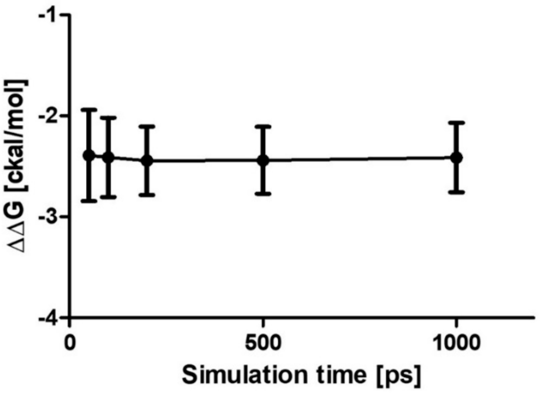

Figure S35. Influence of the simulation time of alchemical transformations on the $\Delta\Delta G_{folding}$ of $PCNA_{A148C}$. $\Delta\Delta G_{folding}$ was calculated from three independent equilibrium trajectories. From these, transition simulations with different length were started. The mean and standard deviation of $\Delta\Delta G_{folding}$ are plotted against the simulation time for each transition simulation.
